# Integrated E-Nose, E-Tongue, HS–GC–IMS, and HS–SPME–GC–MS with Multivariate Statistics for Flavor Discrimination and Volatile Marker Screening of American Ginseng from Different Origins

**DOI:** 10.3390/foods15214011

**Published:** 2026-11-09

**Authors:** Yuchen Yao, Yan Gao, Junqing Li, Chengxia Yin, Bonian Zhao, Lu Liu

**Affiliations:** 1 Institute of Pharmacy, Shandong University of Traditional Chinese Medicine, Jinan 250355, China; yychen67@163.com (Y.Y.); 60230003@sdutcm.edu.cn (Y.G.); 2 Shandong Key Laboratory of Digital Traditional Chinese Medicine, Shandong University of Traditional Chinese Medicine, Jinan 250355, China; 3 Dongying Institute for Food and Drug Control, Dongying 257091, China; ljqdys@163.com (J.L.); dyfda8087851@163.com (C.Y.)

**Keywords:** American ginseng, origin traceability, E-nose, E-tongue, HS–GC–IMS, HS–SPME–GC–MS

## Abstract

American ginseng (*Panax quinquefolius* L.) from different origins exhibits considerable quality variation, yet the flavor differences among them remain poorly characterized. In this study, samples from Shandong, Jilin, and Canada were analyzed by integrating an electronic nose (E-nose) and electronic tongue (E-tongue) with headspace gas chromatography–ion mobility spectrometry (HS–GC–IMS), headspace solid-phase microextraction–gas chromatography–mass spectrometry (HS–SPME–GC–MS), and multivariate statistical methods. The E-nose and E-tongue, combined with partial least squares discriminant analysis (PLS–DA), effectively distinguished the American ginseng samples according to their assigned geographical origins, and key discriminating sensors and taste indicators were screened. HS–GC–IMS and HS–SPME–GC–MS provided complementary advantages and jointly confirmed that terpenes are the main volatile components of American ginseng. Using orthogonal partial least-squares discriminant analysis (OPLS–DA) with variable importance in projection (VIP) > 1, 37 and 42 candidate discriminants were screened by HS–GC–IMS and HS–SPME–GC–MS, respectively, with camphene and α-phellandrene identified by HS–GC–IMS and ginsinsene by HS–SPME–GC–MS as key terpenoid markers. Correlation analyses revealed that these volatile compounds underpinned E-nose-based origin-related aroma discrimination. A coupling association existed between E-tongue and E-nose signals. An electronic sensory–chemometric evaluation system was thus established to screen volatile characteristic markers, providing a basis for flavor fingerprint construction and origin tracing of American ginseng.

## 1. Introduction

American ginseng (*Panax quinquefolius* L.) contains abundant bioactive constituents, including ginsenosides [1], polysaccharides [2], and amino acids [3]. These characteristic components endow the herb with multiple pharmacological properties, including cardioprotective [4,5], anti-fatigue [6,7], immunomodulatory [8,9], hypoglycemic, antimicrobial [10], and anti-inflammatory activities [11,12], making it widely used in health care, functional foods, and the pharmaceutical industry. American ginseng originated in Montreal, Vancouver Mountains, Quebec and the eastern part of the United States. It was introduced to China in the 1870s. Now, it is mainly cultivated in Shandong and Jilin provinces, and its products are widely consumed around the world. Owing to distinct climatic conditions, soil properties, and cultivation practices across growing regions, the accumulation of secondary metabolites and the sensory quality of American ginseng vary significantly among geographical origins, resulting in pronounced regional quality differentiation [13].

Flavor is one of the most critical sensory attributes for evaluating the overall quality, commodity value, and consumer acceptance of American ginseng [14]. The unique aroma and taste of ginseng are determined by endogenous volatile compounds and soluble taste-active substances. Nevertheless, the volatile profile of American ginseng is characterized by low abundance, complex composition, and trace characteristic odor components, which pose challenges for efficient extraction and accurate identification [15]. At present, most studies on American ginseng focus on the isolation and quantitative detection of ginsenosides and polysaccharides [16]. In contrast, systematic investigations of flavor profiles and origin-based volatile differences in American ginseng remain insufficient, and the characteristic aroma-active compounds responsible for geographical discrimination remain unclear [17]. The lack of comprehensive flavor information severely restricts quality evaluation, geographical traceability, and standardized classification of American ginseng.

Bionic sensory technologies, including electronic nose (E-nose) and electronic tongue (E-tongue), can rapidly and objectively characterize the overall aroma and taste attributes of herbal samples without complex pretreatment, providing intuitive sensory differentiation results [18,19]. However, these techniques cannot identify the specific volatile chemical components responsible for flavor differences. Headspace solid-phase microextraction–gas chromatography–mass spectrometry (HS–SPME–GC–MS) offers superior qualitative and quantitative capabilities for volatile constituents, whereas headspace gas chromatography–ion mobility spectrometry (HS–GC–IMS) exhibits high sensitivity, low detection limits, and excellent performance in identifying trace and low-molecular-weight volatile compounds. Nevertheless, each technique alone has inherent limitations: HS–SPME–GC–MS is less sensitive to highly volatile low-molecular-weight compounds, whereas HS–GC–IMS shows restricted qualitative capability for semi-volatile and weakly polar constituents. The combined application of GC–MS and GC–IMS can effectively complement their respective detection advantages, expand the coverage of volatile components, and improve the accuracy and completeness of flavor substance identification [20,21,22].

In this study, the overall sensory flavor characteristics of American ginseng from different major producing regions were comprehensively characterized using the E-nose and E-tongue. Furthermore, GC–IMS and GC–MS were applied to analyze volatile organic compound profiles and screen differential aroma-active components. Combined with multivariate statistical analysis, the regional flavor differentiation patterns of American ginseng were explored. This study aimed to clarify the chemical basis of geographical flavor differences and provide a scientific reference for the establishment of flavor fingerprints and origin traceability of American ginseng.

## 2. Materials and Methods

### 2.1. Material Sources

A total of 60 batches of American ginseng were prepared for analysis. Twenty batches were freshly harvested from standardized planting bases in Shandong Province (SD, China), another 20 batches were collected from cultivation bases in Jilin Province (JL, China), and the remaining 20 batches from Canada (CAN) were purchased through overseas commercial channels. All botanical samples were rigorously verified and authenticated by Professor Bo-nian Zhao at Shandong University of Traditional Chinese Medicine. Each batch was pulverized using a high-speed multifunctional pulverizer (DFY-500D, Linda Machinery, Taizhou, Zhejiang, China) and passed through a 65-mesh sieve (≤250 μm). The powdered samples were placed in sealed bags and stored at 4 °C for subsequent analysis. In this study, a batch was defined as one independent lot of raw roots obtained from a single farm (SD, JL) or a single commercial lot (CAN). The 60 batches were sourced from 8 independent sources (3 farms in SD, 4 farms in JL, and 1 supplier in CAN).

### 2.2. E-Nose Analysis

Electronic nose analysis was performed using the PEN3 electronic nose system (AIRSENSE Analytics GmbH, Schwerin, Germany). The PEN3 system was equipped with 10 metal oxide sensors (W1C, W5S, W3C, W6S, W5C, W1S, W1W, W2S, W2W, and W3S) for volatile profiling. The response characteristics of these ten metal-oxide sensors are summarized in Table S2. A 1.0 g portion of powder was accurately weighed into a 20 mL headspace vial, and the vial was sealed with double-layer film. After heating in an 80 °C water bath for 15 min to allow the headspace gas to reach equilibrium and facilitate volatile enrichment, the headspace gas was pumped at a flow rate of 400 mL/min. Each measurement lasted 120 s, followed by a 120 s system cleaning cycle. The average signal collected in the final 3 s was defined as one replicate.

### 2.3. E-Tongue Analysis

The taste characteristics of American ginseng samples from three origins were evaluated using an electronic tongue system (SA402B, Smart Sensor Technology Co., Tokyo, Japan). The SA402B taste analysis system is equipped with multiple sensor electrodes for basic tastes and corresponding aftertastes, and their response properties are listed in Table S3. A 0.5 g portion of powder was transferred into a conical flask containing 150 mL of ultrapure water and extracted using an ultrasonic device for 45 min (power: 180 watts, frequency: 40 kHz) [23]. Subsequently, the extract was centrifuged at 8500 r/min for 15 min, and the supernatant was collected and filtered as the test sample solution. Before each measurement, the electronic tongue system underwent a 90 s cleaning of the positive and negative electrodes; the reference solution was washed twice, and the reference electrode was rinsed twice for 120 s each (240 s in total). After cleaning, the sample was tested for 30 s, with data collected once per second. The data collected at 30 s were selected as the measurement value [24].

### 2.4. HS–GC–IMS Analysis

Volatile organic compounds in American ginseng samples were detected using HS–GC–IMS (FlavourSpec^®^ flavor analyzer, G.A.S. Company, Dortmund, Germany). Powdered American ginseng samples from different batches (2.0 g each) were accurately weighed and placed into 20 mL headspace vials. For each batch, 500 µL of 10 ppm 2-methyl-3-heptanone was added as an internal standard to determine the volatile components in American ginseng. Samples were incubated at 85 °C with shaking at 500 rpm for 30 min; the injection needle temperature was 90 °C, and the injection volume was 500 µL. The chromatographic column was an MXT-WAX polar column (15 m × 0.53 mm, 1.0 μm, Restek, Bellefonte, PA, USA); the column temperature was 65 °C, and the IMS detector temperature was 45 °C; carrier/drift gas was high-purity nitrogen (mass fraction ≥ 99.999%); and the drift gas flow rate was 75 mL/min. The gas-phase carrier flow rate was programmed as follows: initial flow of 2 mL/min, maintained for 5 min; linearly increased to 10 mL/min within 5 min; linearly increased to 75 mL/min from 10 to 30 min; linearly increased to 100 mL/min from 30 to 40 min; and maintained for 10 min (total analysis time: 50 min). GC–IMS qualitative analysis was performed using C4-C9 n-ketones as calibration standards, combined with the software-embedded NIST2020 and IMS Library 1.5 databases, by comparing the retention index (RI) and drift time (DT) of volatile compounds in the samples. Semi-quantitative analysis of volatile compounds was performed by dividing the peak area of the target compound by that of 2-methyl-3-heptanone as the internal standard [25,26].

### 2.5. HS–SPME–GC–MS Analysis

HS–SPME–GC–MS was applied for the qualitative identification and quantitative analysis of volatile compounds in American ginseng samples. For sample preparation, 0.5 g of American ginseng powder was transferred into a 20 mL headspace vial, followed by the addition of 200 µL of 10 ppm 2-methyl-3-heptanone (prepared in methanol) as the internal standard, yielding an internal standard amount of 2 μg for each sample. Although the introduction of 200 µL of methanolic solution may slightly modify headspace partitioning, an identical volume was spiked into all samples. This consistent treatment ensures that the solvent-related effect is uniform across groups and does not interfere with inter-origin comparisons. Samples were incubated within a 70 °C water bath for 30 min. A 2-cm SPME fiber coated with divinylbenzene/carboxen/polydimethylsiloxane (DVB/CAR/PDMS, 50 µm film thickness; Supelco, Bellefonte, PA, USA) was used. The fiber was conditioned in the GC injector at 250 °C for 60 min prior to first use and at 250 °C for 10 min before each analysis. Subsequently, the SPME fiber was inserted into the headspace of the vial for 5-min extraction. The fiber was then subjected to thermal desorption inside the GC injector port at 250 °C for 5 min. The 5-min extraction time was selected as a compromise between sensitivity and throughput; the repeatability of the extraction was verified by triplicate analyses of a pooled quality-control sample, with relative standard deviations of 2% for the principal marker compounds. GC–MS analysis was performed on a Trace 1600 gas chromatograph coupled to a TSQ 9610 mass spectrometer (Thermo Fisher Scientific, Waltham, MA, USA). Chromatographic separation was performed on a TG-5 SIL MS medium-polar capillary column (30 m × 0.25 mm, 0.25 µm, Thermo Scientific, USA). The inlet temperature was maintained at 250 °C, and high-purity helium (99.99%) served as the carrier gas with a constant flow of 1 mL/min. The oven temperature gradient was set starting at 40 °C with a 2 min hold, ramped to 120 °C at 6 °C/min and kept isothermal for 15 min, subsequently raised to 150 °C at 10 °C/min with a 2 min hold, and finally heated to 250 °C at 20 °C/min, followed by another 2 min holding stage. The total time was 42.3 min; the split ratio was 5:1; ion source temperature was 230 °C; transfer line temperature was 250 °C; electron ionization (EI) energy was 70 eV; and the ion scan range was 30–450 m/z. Blank runs (empty vials subjected to the same SPME procedure) were performed every 10 samples to monitor carryover, and no significant carryover was observed. Compound identification was implemented by matching mass spectra against the NIST17 library. Compounds were identified when both the similarity index (SI) and reverse similarity index (RSI) exceeded 800 [27]. The peak area of each target compound was divided by that of 2-methyl-3-heptanone, which served as the internal standard, for semi-quantitative analysis of the volatile compounds.

### 2.6. Statistical Analysis

All measurements were conducted in triplicate unless noted otherwise. The HS–GC–IMS raw datasets were handled via VOCal software v.0.4.03 (G.A.S. Company for Analytical Sensor Systems Ltd., Dortmund, Germany) together with its two auxiliary modules, Reporter and Gallery Plot. Origin 2024 (OriginLab Corporation, Northampton, MA, USA) was utilized for graphical visualization. Principal component analysis (PCA), partial least squares discriminant analysis (PLS–DA), and orthogonal partial least-squares discriminant analysis (OPLS–DA) were implemented in PyCharm 2026.1 (JetBrains s.r.o., Prague, Czech Republic). PCA is an unsupervised dimensionality-reduction algorithm. PC1 corresponds to the principal component explaining the largest proportion of the total variance of the original dataset, while PC2 is the orthogonal second principal component that accounts for the second-largest variance. PCA scores are obtained via linear transformation of the standardized raw sensor signal matrix; each principal component is a weighted linear combination of all original variables. PLS–DA is a supervised discriminant method that maximizes the covariance between the predictor matrix and the categorical group label matrix. OPLS–DA is an extended form of PLS–DA. It separates systematic variation within the dataset into one predictive component associated with group discrimination and orthogonal components unrelated to sample classification. Model performance was evaluated using R2X, R2Y and Q2, and permutation tests were performed to avoid overfitting. Hierarchical clustering heatmaps were generated using ChiPlot (https://www.chiplot.online/, accessed on 23 September 2026).

## 3. Results

### 3.1. E-Nose Analysis

**Figure 1:**
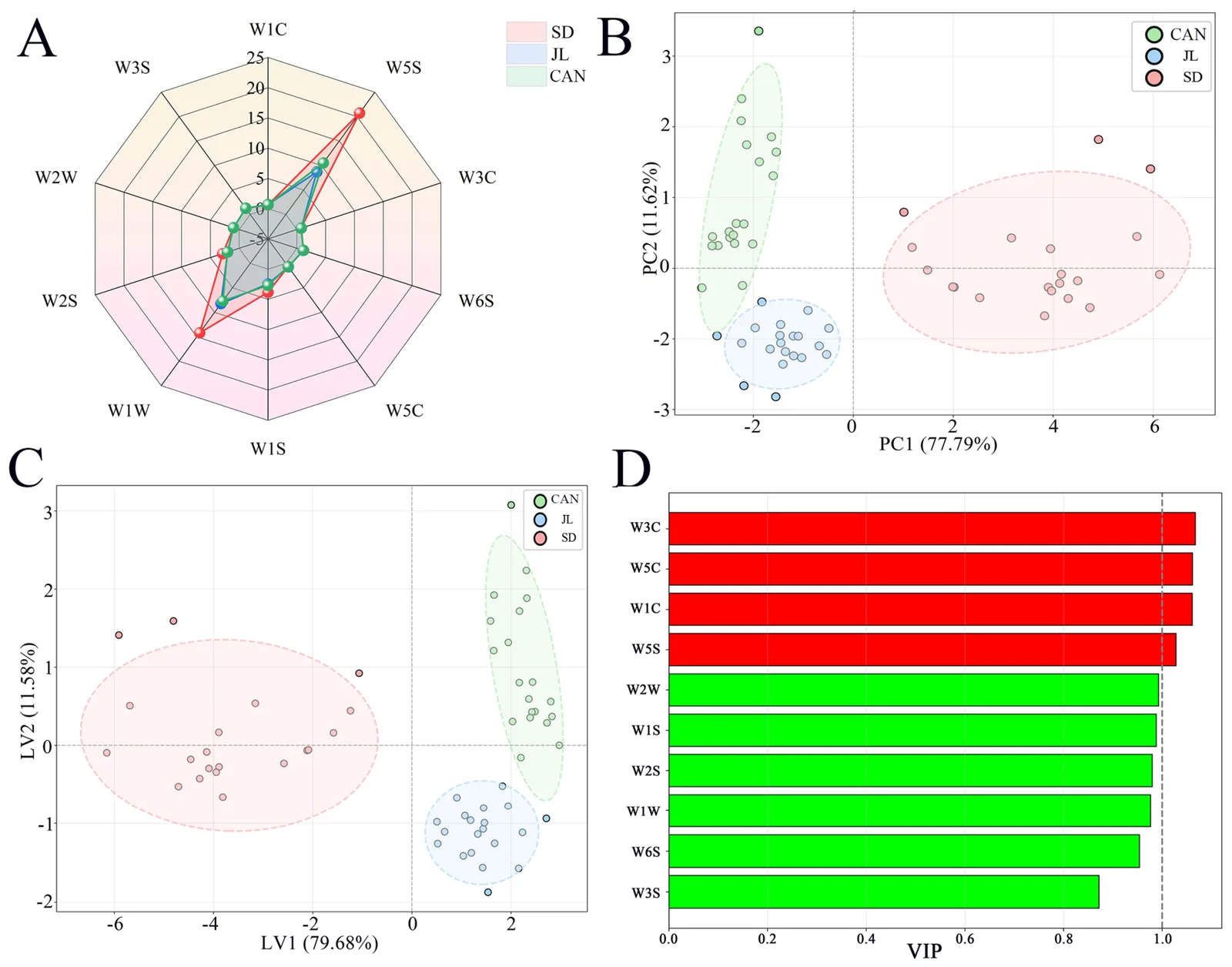
E-nose analysis of American ginseng samples from three geographical origins (SD, Shandong, China; JL, Jilin, China; CAN, Canada). (**A**) Sensor response radar plot; (**B**) PCA score plot based on the E-nose sensor responses; (**C**) PLS–DA discrimination plot based on the E-nose sensor responses; (**D**) VIP variable importance bar chart. Red bar (VIP > 1) indicates that the variable has high importance in the OPLS–DA model. This is a model-dependent importance measure and does not represent a test of statistical significance.

E-nose technology enables non-destructive, high-throughput characterization of the overall volatile odor profile of samples. In this study, an electronic nose equipped with 10 sensors was used to characterize the volatile odor profiles of American ginseng samples from three geographical origins. The radar chart derived from the electronic nose dataset (Figure 1A) revealed generally similar contour patterns across the three groups, indicating a shared fundamental pool of volatile constituents among all samples. Sensors W5S, W1W, and W1S exhibited the most prominent signal responses. According to the detection targets of the PEN3 sensors specified by the manufacturer (Table S2), W5S shows broad-range sensitivity with a strong response to nitrogen oxides, W1W responds to sulfur-containing compounds and terpenes, and W1S shows broad-range sensitivity with a strong response to methane and methyl derivatives. Therefore, the overall odor profile of the samples was tentatively attributed to nitrogen oxides, sulfur-containing compounds, methyl derivatives, and related substances. It should be noted that the E-nose provides only a nonspecific response pattern and cannot identify individual volatile compounds; this tentative assignment was therefore further examined by HS–GC–IMS and HS–SPME–GC–MS. The SD group exhibited the highest overall odor response intensity, with markedly higher signals on sensors W5S and W1W than the JL and CAN groups; distinct sensor response magnitudes were also observed between JL and CAN samples.

PCA was performed on the electronic nose dataset (Figure 1B). The first two principal components, PC1 and PC2, accounted for 77.79% and 11.62% of the total variance respectively, with a cumulative explanatory rate of 89.41%, capturing the dominant variation in odor attributes. Within the PCA score plot, the SD samples formed a distinct cluster well separated from the other two groups, indicative of divergent aromatic signatures, consistent with the sensor response trends illustrated in the radar chart. Despite a partial similarity in olfactory features between JL and CAN samples, the overall volatile fingerprints of American ginseng varied considerably by cultivation origin, enabling geographical discrimination based on these odor patterns. A PLS–DA model based on electronic nose data achieved robust differentiation of American ginseng from the three producing regions (Figure 1C), and a 200-times permutation test confirmed that the model was not overfitted (Figure S1A). The first two latent variables (LV1 and LV2) explained a cumulative variance of 91.26% (79.68% for LV1 and 11.58% for LV2), sufficiently representing intergroup olfactory differences. Samples from SD, JL and CAN formed three discrete clusters in the score plot, with tight intra-group aggregation and no inter-group overlap. This result verified significant inter-origin differences in volatile profiles and confirmed the excellent discriminative performance of the PLS–DA model for accurate geographical traceability of American ginseng. The distribution patterns of the three groups differed between the PCA and PLS–DA score plots. In the PCA score plot (Figure 1B), the SD samples were clearly separated, whereas the JL and CAN samples partially overlapped, because PCA is an unsupervised method that projects the total variance without using origin information, and the overall odor variance is dominated by within-group and inter-individual variation. In contrast, PLS–DA (Figure 1C) is a supervised method that uses origin labels to maximize inter-group separation, so the three groups formed discrete clusters with no overlap. This comparison indicates that the inter-origin differences, although present, are partially masked by within-group variation in the unsupervised analysis. Nevertheless, the electronic nose cannot qualitatively identify individual volatile compounds; therefore, complementary gas chromatography-based analysis is required for further compositional elucidation. The variable importance in projection (VIP) results of PLS–DA are shown in Figure 1D, where each bar represents a different electronic nose sensor. Red and green bars correspond to the VIP values of the sensors for the two sample groups, and the dashed line represents the critical threshold of VIP = 1. Sensors W3C, W5C, W1C, and W5S had VIP values greater than 1 and were key sensors for sample discrimination, whereas the remaining sensors had VIP values below 1 and contributed relatively little to discrimination. Combined with the permutation test results, the PLS–DA model was stable and reliable and could effectively screen electronic nose sensors that contribute significantly to sample flavor differences.

### 3.2. E-Tongue Analysis

**Figure 2:**
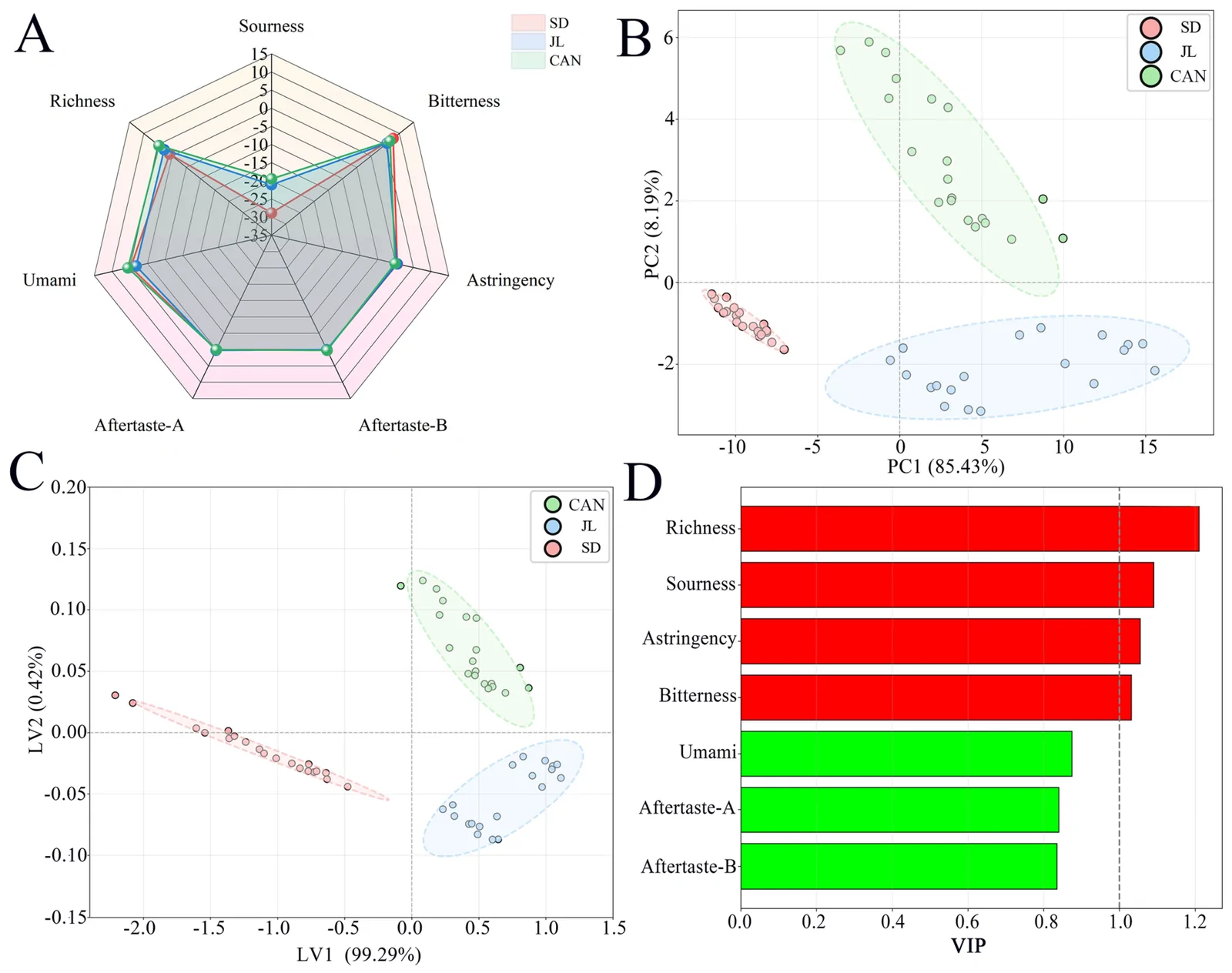
E-tongue analysis of American ginseng samples from three geographical origins. (**A**) Sensor response radar plot; (**B**) PCA score plot based on the E-tongue taste signals; (**C**) PLS–DA discrimination plot based on the E-tongue taste signals; (**D**) VIP variable importance bar chart. Red bar (VIP > 1) indicates that the variable has high importance in the OPLS–DA model. This is a model-dependent importance measure and does not represent a test of statistical significance.

E-tongue technology permits objective quantification of taste attributes. In this study, the electronic tongue was adopted to explore variations in the taste profiles of American ginseng samples from different geographical origins. The basic sensory attributes were displayed in a radar chart (Figure 2A), which revealed that richness, sourness, bitterness, and astringency formed the core taste characteristics of American ginseng. Remarkable inter-group variations were detected in umami, aftertaste-A, and aftertaste-B. The SD group exhibited the weakest responses for bitterness and astringency, the CAN group showed the most prominent responses for richness and sourness, and the JL group maintained moderate values across all taste indicators. Richness and sourness were markedly higher in CAN American ginseng relative to the other two groups. Except for the JL group, the SD and CAN groups shared an identical rank order of taste attribute intensities, differing only in the magnitude of each response.

To further elucidate taste variations, PCA was performed on the electronic tongue dataset (Figure 2B). Complete separation among the three groups was achieved in the two-dimensional score plot. PC1 and PC2 contributed 85.43% and 8.19% of the total variance, respectively, with a cumulative variance explanation rate of 93.62%. A shorter clustering distance indicates higher similarity in taste fingerprints: SD samples clustered on the left, JL samples in the lower-right quadrant, and CAN samples in the upper-left area. No obvious overlap was observed among the three groups, directly verifying distinct taste fingerprints of American ginseng from the three origins. These clustering results were highly consistent with the variation trend of taste intensities in the radar chart. A PLS–DA model was further constructed to verify the capacity of the electronic tongue for geographical traceability of American ginseng. The first two latent variables, LV1 and LV2, together explained up to 99.71% of the total variance, indicating excellent discriminative efficacy and statistically significant origin-induced differences in the taste properties of American ginseng (Figure 2C). VIP analysis based on the PLS–DA model (Figure 2D) revealed that richness, sourness, astringency, and bitterness had VIP values greater than 1, serving as core taste discriminant markers for distinguishing American ginseng from different habitats. This finding was highly consistent with the radar chart observation that these four parameters displayed the most pronounced inter-group differences. Similarly, the PCA score plot of the E-tongue data (Figure 2B) showed complete separation of the three groups, whereas the PLS–DA score plot (Figure 2C) gave a more compact and better-defined clustering with a higher cumulative variance (99.71%). The difference in distribution patterns reflects the fact that PCA describes the overall taste variance in an unsupervised manner, whereas PLS–DA emphasizes the variance associated with origin; the consistency of the grouping trends between the two analyses confirms that taste differences among origins are robust.

Effective geographical discrimination of American ginseng was achieved solely based on the taste signals acquired in this study. Collectively, these findings validate the potential of electronic sensory technology for rapid and reliable geographical traceability of American ginseng.

### 3.3. Analysis of Volatile Organic Compounds (VOCs) in American Ginseng Based on HS–GC–IMS

**Figure 3:**
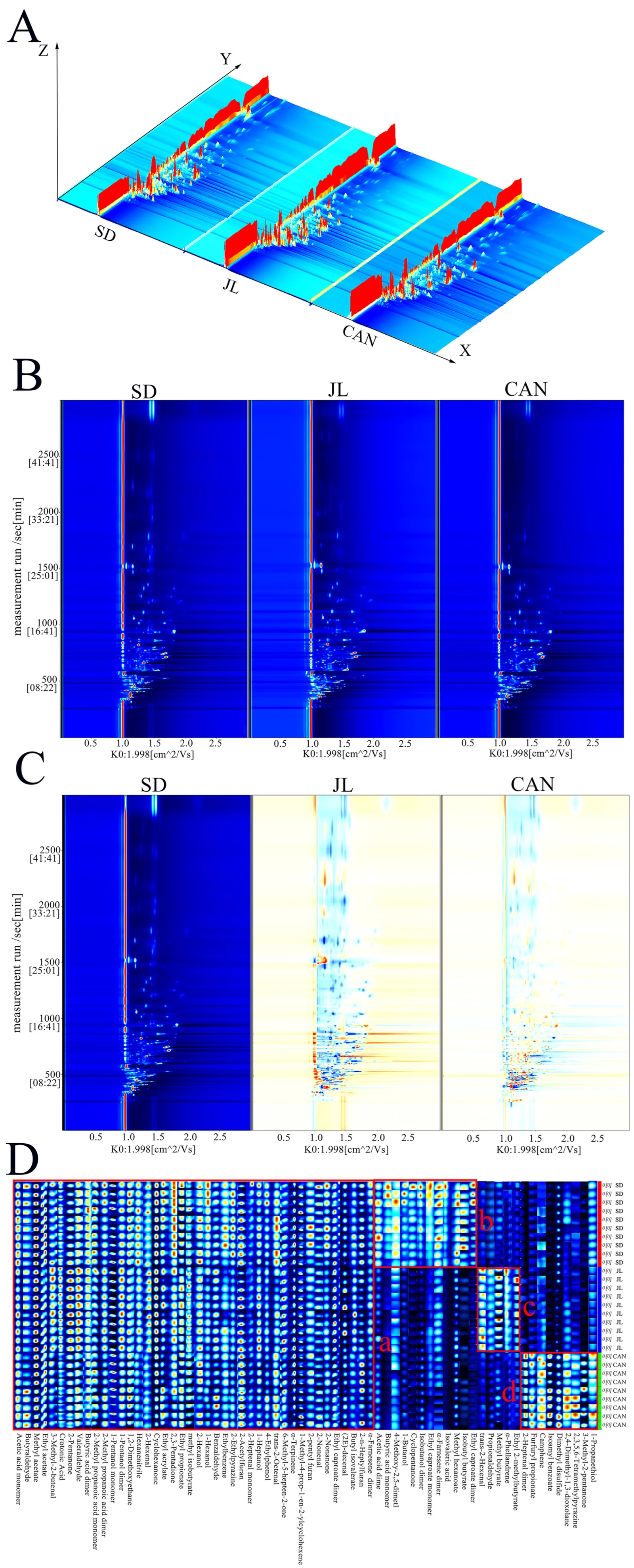
VOC profile information obtained by HS–GC–IMS for American ginseng samples of different geographical origins. (**A**) Three-dimensional topology plots. (**B**) Two-dimensional topology plots. (**C**) Comparative difference topology plots. (**D**) VOC fingerprint of American ginseng samples. The colored boxes a, b, c and d denote four characteristic regions with distinct volatile compound profiles. Specifically, red box a indicates volatile compounds common to all three origins (e.g., α-farnesene and acetic acid); red box b indicates compounds with high contents unique to SD samples (e.g., isobutyl butyrate); red box c indicates compounds with high contents unique to JL samples (e.g., α-phellandrene); and red box d indicates compounds with high contents unique to Canadian samples (e.g., 2-methylpropanoic acid dimer).

HS–GC–IMS was used to analyze VOCs in American ginseng samples collected from three regions. HS–GC–IMS expanded upon the findings from the electronic nose and electronic tongue, thereby enhancing the profiling of volatiles in American ginseng. In total, 60 compounds (corresponding to 68 volatile organic compound signal peaks) were identified (Table S2). Some compounds existed in monomeric and dimeric forms, including 13 esters, 11 aldehydes, 7 ketones, 6 alcohols, 5 terpenes, 5 carboxylic acids, 4 furans, 2 pyrazines, 2 ethers, 2 sulfur-containing compounds, 1 aromatic hydrocarbon, 1 nitrile, and 1 phenol. Three-dimensional comparison of American ginseng samples from the three producing regions was performed using the built-in analysis software of the HS–GC–IMS instrument (Figure 3A). The three-dimensional spectra enabled intuitive comparison of volatile components among American ginseng samples from different origins in terms of peak position, peak number, and peak intensity. The *X*-axis represented ion drift time, the *Y*-axis represented GC retention time, and the *Z*-axis represented ion reaction peak intensity for quantification. Comparison of the three-dimensional spectra revealed slight differences in volatile components among American ginseng samples from different producing regions, despite similar peak intensities. The three-dimensional spectra were converted into two-dimensional top-view contour plots for subsequent analysis. The two-dimensional plots used a blue background, and the normalized reactive ion peak (RIP) was located near an abscissa of 1.0, marked with a vertical red line. Each signal point on either side of the RIP represented one volatile component, and the signal color reflected its relative peak intensity; the color gradually deepened from white to red, indicating an increase in relative abundance. As illustrated in Figure 3B, the majority of VOC responses were detected over the retention period of 200–1500 s and drift time interval of 1.0–2.0 s, suggesting similar VOC compositions among American ginseng samples from different origins. Figure 3C shows that when SD samples were used as the reference, JL samples exhibited the most pronounced differences, with numerous differential spots and marked variations in peak intensity. With SD samples as the reference, blue regions in the two-dimensional spectra of samples from the other two origins indicated lower signal intensity than that of SD samples, white regions indicated comparable intensity, and red regions indicated higher intensity. Blue and red signal regions were also observed to varying degrees in CAN samples. These results indicated that American ginseng from the three origins shared a common pool of volatile components while possessing distinguishable spectral features, providing a basis for subsequent origin discrimination and screening of differential volatile components.

To explore the specific volatile variations among American ginseng from distinct producing areas, a fingerprint plot encompassing all components from the HS–GC–IMS two-dimensional spectra was constructed (Figure 3D). In this visualization, individual samples are arranged as rows displaying their complete volatile signal profiles, whereas the columns correspond to the signal intensities of identical volatile components across the various samples. Red box a indicates volatile compounds common to American ginseng from all three origins (e.g., α-farnesene and acetic acid). Red box b indicates volatile compounds with high contents unique to SD samples (e.g., isobutyl butyrate). Red box c indicates volatile compounds with high contents unique to JL samples (e.g., α-phellandrene). Red box d indicates volatile compounds with high contents unique to Canadian samples (e.g., 2-methylpropanoic acid dimer). These differential compounds may serve as potential characteristic variables for discriminating the three producing regions. Overall, the fingerprint plot clearly reflected similarities and differences in volatile components of American ginseng from the three origins, providing intuitive evidence for subsequent multivariate statistical analysis. For clearer comparison, the distribution of volatile organic compounds among all detected volatile components was analyzed for each origin.

**Figure 4:**
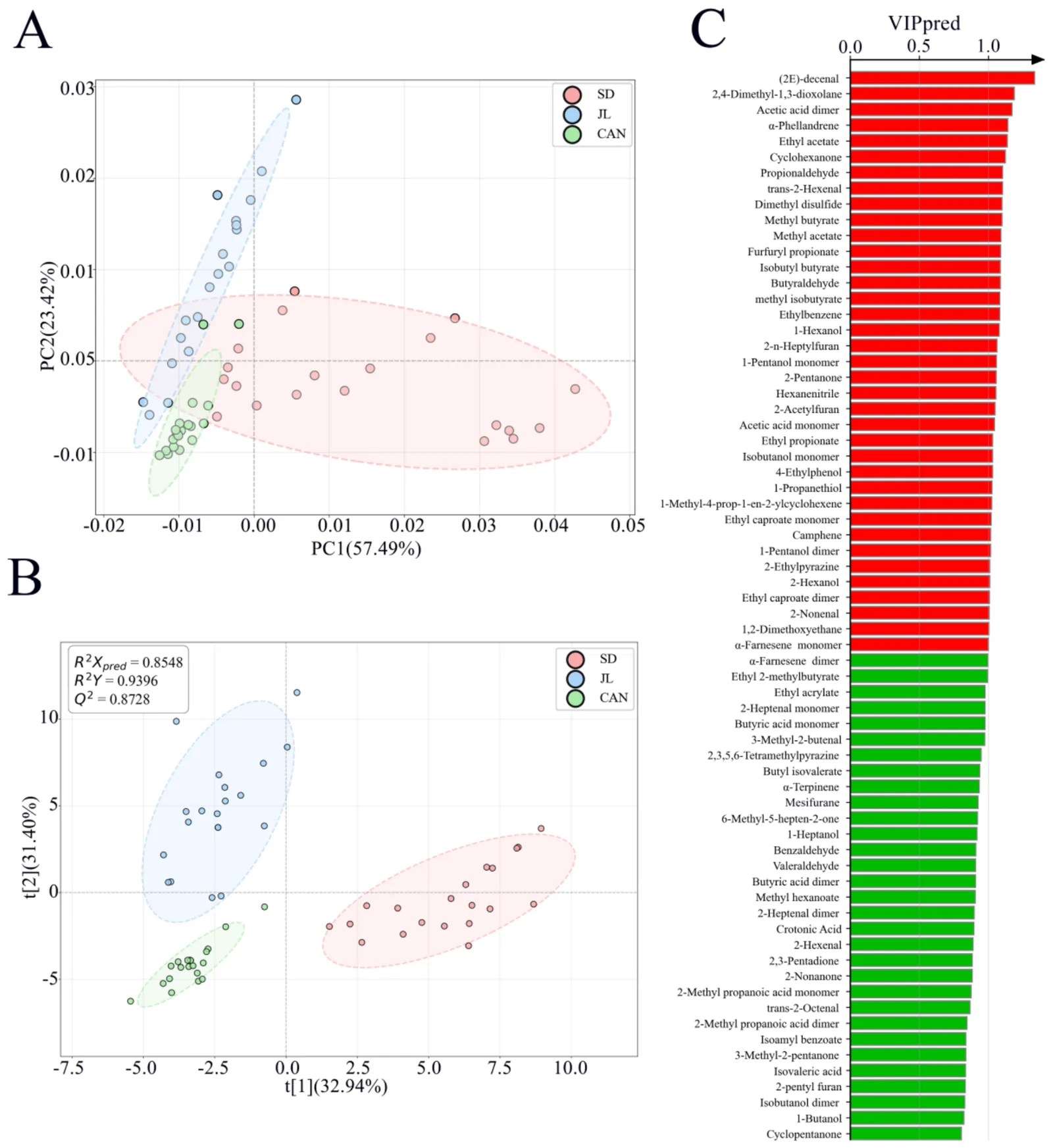
HS–GC–IMS evaluation of American ginseng specimens obtained from various geographical sources. (**A**) PCA score plot. (**B**) OPLS–DA score plot. (**C**) VIP variable importance bar chart. Red bar (VIP > 1) indicates that the variable has high importance in the OPLS–DA model. This is a model-dependent importance measure and does not represent a test of statistical significance.

PCA was performed using the content of each volatile component as a variable to comprehensively evaluate differences in the VOC profiles of American ginseng from the three origins. The PCA results are shown in Figure 4A. The combined explained variance of PC1 and PC2 amounted to 80.91%, indicating that these first two principal components captured the majority of the variance in the VOC dataset. In the PCA score plot, the samples from the three origins exhibited a tendency to separate. Specifically, SD samples were clearly distinguished from the other two groups, implying notable differences in their overall volatile profiles. JL samples were positioned near the other group, reflecting similarities in both volatile composition and relative abundance between these two sets.

To further improve discrimination of American ginseng samples from different origins and to screen key volatile components contributing to sample classification, supervised OPLS–DA was carried out based on concentration datasets of 86 VOC peak signals. The OPLS–DA model was constructed by adopting volatile constituent concentrations as predictors and growing locality as the response factor; the analytical outcomes are displayed in Figure 4B. American ginseng specimens from the three origins achieved satisfactory grouping and segregation within the model, proving that their VOC fingerprints could well distinguish inter-location discrepancies. The calculated model metrics R^2^X = 0.8548, R^2^Y = 0.9396, and Q^2^ = 0.8728 reflected reliable fitting quality and predictive capacity. Altogether, 200 permutation validations were implemented to verify the stability and dependability of this OPLS–DA model (Figure S1C). The permutation testing yielded intercepts for the R^2^ and Q^2^ regression lines of 0.236 and –0.850, respectively. The Q^2^ intercept fell below zero, and the Q^2^ values of the permuted models were typically lower than those of the original model, which indicates the absence of obvious overfitting and confirms the model’s strong robustness and statistical dependability. Consequently, this OPLS–DA model was appropriate for the ensuing screening of differential volatile constituents.

**Figure 5:**
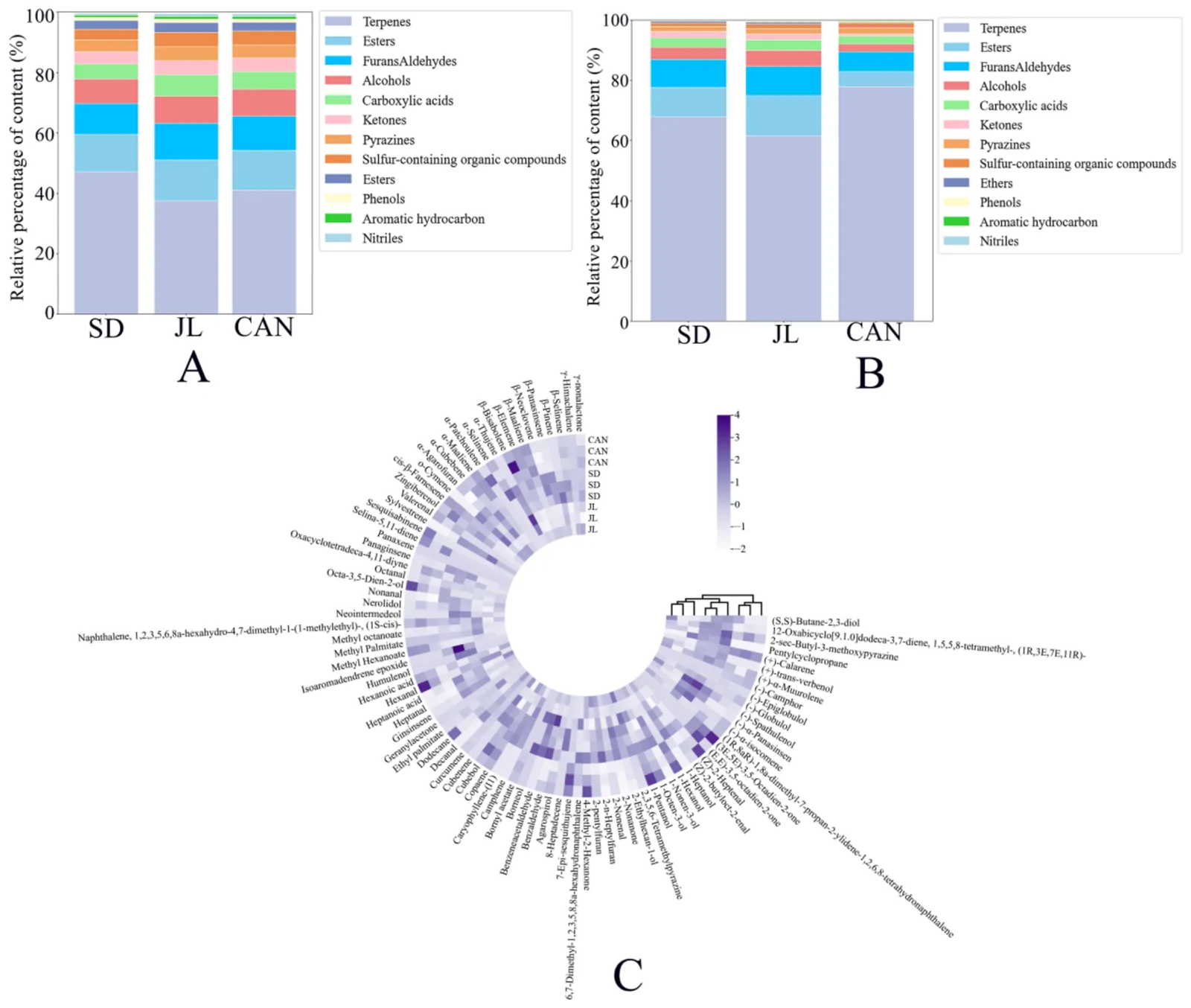
(**A**) Percentage stacked histogram of HS–GC–IMS volatile components. (**B**) Percentage stacked histogram of HS–SPME–GC–MS volatile components. (**C**) Hierarchical clustering heatmap of HS–SPME–GC–MS data: the color represents the relative abundance of each compound; darker purple indicates higher relative abundance.

VIPpred was employed to assess how much each variable contributed to the classification performance of the OPLS–DA model. A higher VIP value signified a stronger contribution of the corresponding volatile compound toward differentiating American ginseng samples from various origins. In the present study, a screening threshold of VIPpred > 1.0 was established, resulting in the identification of 37 potential differential volatile components that substantially contributed to distinguishing the three origins (Figure 4C). The 37 screened potential differential volatile components were (2E)-decenal, 2,4-dimethyl-1,3-dioxolane, acetic acid dimer, α-phellandrene, ethyl acetate, cyclohexanone, propionaldehyde, trans-2-hexenal, dimethyl disulfide, methyl butyrate, methyl acetate, furfuryl propionate, isobutyl butyrate, butyraldehyde, methyl isobutyrate, ethylbenzene, 1-hexanol, 2-n-heptylfuran, 1-pentanol monomer, 2-pentanone, hexanenitrile, 2-acetylfuran, acetic acid monomer, ethyl propionate, isobutanol monomer, 4-ethylphenol, 1-propanethiol, 1-methyl-4-(prop-1-en-2-yl)cyclohexene, ethyl caproate monomer, camphene, 1-pentanol dimer, 2-ethylpyrazine, 2-hexanol, ethyl caproate dimer, 2-nonenal, 1,2-dimethoxyethane, and α-farnesene monomer. These compounds can be regarded as key markers for further research on origin identification and quality evaluation of American ginseng. Figure 5A presents the relative contents of volatile organic compounds in samples from each origin. Terpenes, esters, furans, and aldehydes were the major flavor contributors, accounting for more than 50% of total volatile substances, consistent with previous reports [28].

### 3.4. Analysis of Volatile Organic Compounds (VOCs) in American Ginseng Based on HS–SPME–GC–MS

Volatile constituents from American ginseng specimens of distinct geographical sources were subjected to qualitative and semi-quantitative measurement via HS–SPME–GC–MS. The purpose was to cross-validate the results obtained by GC–IMS and to establish a more comprehensive flavor profile. In total, 89 volatile organic analytes were characterized, including 31 sesquiterpenes, 17 alcohols, 11 aldehydes, 7 alkanes/cycloalkenes, 6 esters, 6 monoterpenes, 5 ketones, 2 carboxylic acids, 2 furans, 2 pyrazines, and 1 other heterocyclic compound (Table S3). The histogram of relative VOC category contents among different origins (Figure 5B) showed that sesquiterpenes and alcohols jointly made up over 60% of the total VOC pool across SD, JL and CAN batches and were the dominant VOC categories, constituting the core flavor traits of American ginseng. This trend was consistent with previous reports [29], followed by aldehydes. The relative content of sesquiterpenes in CAN American ginseng reached 77.84%, which was speculated to be related to enhanced terpene secondary metabolism induced by the cool environment at high latitudes. Both analytical methods indicated that terpenes were the dominant volatile components of American ginseng, with consistent variation trends across origins: CAN had the highest relative proportion of terpenes, whereas JL had the lowest. The relative abundances of oxidation- and Maillard-derived components, including aldehydes, alcohols, ketones, and carboxylic acids, were higher in JL samples than in SD and CAN samples. Nevertheless, obvious discrepancies were observed in the relative percentage contents determined by the two methods. The relative proportion of terpenes determined by GC–MS (Figure 5B) was markedly higher than that obtained by HS–GC–IMS (Figure 5A); by contrast, HS–GC–IMS yielded higher proportions of small-molecule polar volatiles such as alcohols, aldehydes, ketones, carboxylic acids, and furans. Such discrepancies originate from detector principles: soft ionization in GC–IMS confers higher sensitivity toward low-molecular-weight polar volatile molecules, whereas hard EI ionization in HS–GC–MS is more suitable for medium-to-large weakly polar terpenoids. Notably, the values shown in the figures represent relative percentages calculated by internal peak area normalization for each instrument. Because response factors vary across instruments, these values can be used only for horizontal comparisons among origins within the same analytical method and cannot be directly applied to compare absolute relative contents across instruments. Based on the volatile component data from HS-GC-MS, a circular clustered heatmap was plotted after data standardization, and hierarchical cluster analysis was performed (Figure 5C). Hierarchical clustering results demonstrated that replicate samples from the same origin clustered together, and samples from the three origins (CAN, SD, and JL) were completely separated, indicating significant differences in volatile fingerprints of American ginseng from the three origins. The heatmap revealed that volatiles could be divided into two major clustering modules: terpenoids exhibited higher abundances in CAN and SD American ginseng and served as characteristic volatile components of these two groups, whereas lipid oxidation products and Maillard reaction-derived components (aldehydes, alcohols, ketones, furans, pyrazines, and short-chain carboxylic acids) were significantly enriched in JL American ginseng. These results were consistent with those from the stacked bar chart, demonstrating more prominent accumulation of secondary oxidation and Maillard reaction products in JL American ginseng.

**Figure 6:**
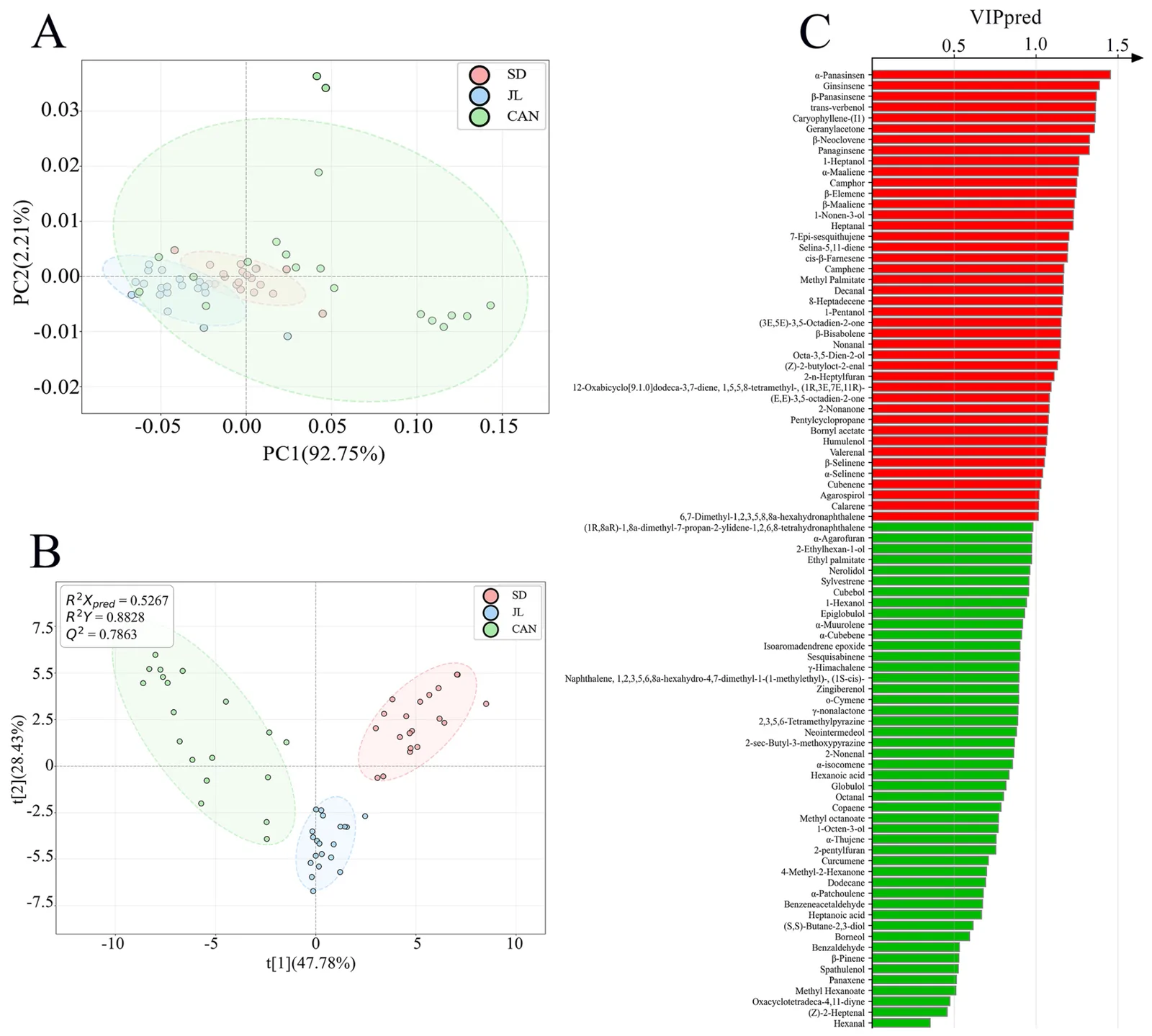
HS–GC–MS profiling of American ginseng specimens collected from various regions. (**A**) PCA score plot. (**B**) OPLS–DA score plot. (**C**) VIP variable importance bar chart. Red bar (VIP > 1) indicates that the variable has high importance in the OPLS–DA model. This is a model-dependent importance measure and does not represent a test of statistical significance.

To identify volatile components with significant content differences capable of discriminating American ginseng from different origins, unsupervised PCA was first performed on all volatile components (Figure 6A). The PCA results showed an initial grouping trend for JL, SD, and CAN samples. JL samples were concentrated on the negative half-axis of PC1, CAN samples on the positive half-axis of PC1, and SD samples in the intermediate region between the two groups; however, partial overlap existed between SD samples and the other two groups. To further amplify intergroup differences and construct an origin discrimination model, OPLS–DA was adopted [30]. The parameters of the OPLS–DA model indicated favorable explanatory capacity and predictive performance. In the OPLS–DA score plot, complete separation of the three groups was achieved without overlap of the 95% confidence ellipses, proving that volatile fingerprints can effectively distinguish American ginseng from the three origins. The evaluation parameters of this model were R^2^X = 0.5267, R^2^Y = 0.8828, and Q^2^ = 0.7863, demonstrating strong predictive ability and satisfactory reliability. As observed in the score plot, the first principal component t [1] (47.78%) and the second principal component t [2] (28.43%) together explained 76.21% of the total variance. Samples from the three origins displayed a clear separation trend in two-dimensional space: the CAN group was located on the left side of the plot, the SD group on the right side, and the JL group mainly clustered in the lower-middle position. The 95% confidence intervals of the three groups were independent, with no overlap. These results showed consistent sample distribution trends between PCA and OPLS–DA. Combined with the stacked bar charts and hierarchical clustering heatmaps, these findings collectively confirmed significant differences in volatile component profiles of American ginseng from different origins, and the OPLS–DA model effectively discriminated them. The regression curves of R^2^ and Q^2^ exhibited an upward trend, and the Q^2^ intercept was less than 0, suggesting that the model passed the permutation test without overfitting (Figure 6B). Therefore, identification of differential compounds based on VIP values from the OPLS–DA model was reliable (Figure S1D). Variables with VIPpred > 1 were marked in red (Figure 6C). Based on the VIP values of each differential volatile compound, a total of 42 standardized differential components were identified, including (−)-α-panasinsen, ginsinsene, β-panasinsene, (−)-trans-verbenol, caryophyllene-(I1), geranylacetone, β-neoclovene, panaginsene, 1-heptanol, α-maaliene, (−)-camphor, β-elemene, β-maaliene, 1-nonen-3-ol, heptanal, 7-epi-sesquithujene, selina-5,11-diene, cis-β-farnesene, camphene, methyl palmitate, decanal, 8-heptadecene, 1-pentanol, (3E,5E)-3,5-octadien-2-one, β-bisabolene, nonanal, octa-3,5-dien-2-ol, (Z)-2-butyloct-2-enal, 2-n-heptylfuran, 12-oxabicyclo[9.1.0]dodeca-3,7-diene, 1,5,5,8-tetramethyl-, (1R,3E,7E,11R)-, (E,E)-3,5-octadien-2-one, 2-nonanone, pentylcyclopropane, bornyl acetate, humulenol, valerenal, β-selinene, α-selinene, cubenene, agarospirol, (+)-calarene, and 6,7-dimethyl-1,2,3,5,8,8a-hexahydronaphthalene.

The differential volatile compounds among the three origins can be tentatively traced to their biosynthetic origins and to environmental factors. Terpenoids, including camphene, α-phellandrene, β-elemene, β-maaliene, and α-farnesene, arise mainly from the mevalonate (MVA) and 2-C-methyl-D-erythritol 4-phosphate (MEP) pathways, and their accumulation is strongly influenced by temperature and light; the higher terpene proportion in CAN samples may therefore reflect the cooler climate at high latitudes, which promotes terpene secondary metabolism. In contrast, aldehydes, alcohols, ketones, furans, pyrazines, and short-chain carboxylic acids are typically derived from lipid oxidation and Maillard reactions, and their enrichment in JL samples may be associated with local cultivation and post-harvest drying conditions. Compounds such as acetic acid, ethyl acetate, and methyl butyrate are common products of esterification and microbial/fermentative metabolism, and their variation may reflect differences in soil and storage conditions among the producing regions.

### 3.5. Correlation Analysis Between Characteristic Volatile Organic Compounds Detected by GC–IMS and GC–MS and the E-Tongue and E-Nose

Correlation analysis allows for the quantification and visualization of potential relationships among multiple variables [31]. In this study, Pearson correlation analysis was performed on characteristic VOCs with VIPpred > 1 (identified by HS–GC–IMS and HS–SPME–GC–MS) along with the E-nose and E-tongue datasets. This analysis combines Pearson correlation and the Mantel test to elucidate covariation among volatile components of American ginseng and to reveal relationships between the overall profile of these candidate discriminants and electronic nose and electronic tongue responses, thereby clarifying the contribution of volatile flavor substances to the aroma and taste attributes of American ginseng.

In the correlation heatmap, color saturation represents the magnitude of correlation (higher color darkness corresponds to stronger correlation values; red denotes positive correlation, while blue stands for negative correlation), and asterisks indicate the significance level. Pearson correlation analysis was performed to evaluate the relationships between the candidate volatile discriminants and the E-nose/E-tongue sensor responses. The observation count for this correlation analysis was *n* = 60 independent batches (with the three analytical replicates averaged for each batch to represent the batch-level value). Prior to analysis, the normality of the data was assessed using the Shapiro–Wilk test, and variables were log-transformed to meet the assumptions of normality and homoscedasticity. The significance of the Pearson correlation coefficients (r) was determined using two-sided tests, and the *p*-values were adjusted for multiple comparisons using the Benjamini–Hochberg (BH) false discovery rate (FDR) procedure (adjusted *p* < 0.05). For the Mantel test, the associations between the volatile discriminant distance matrix and the E-nose/E-tongue distance matrices were calculated. The distance matrices were computed using the Euclidean distance metric based on the same batch-level averaged data. The statistical significance of the Mantel statistic (r) was assessed using 999 permutations. All analyses were performed using ChiPlot online platform.

**Figure 7:**
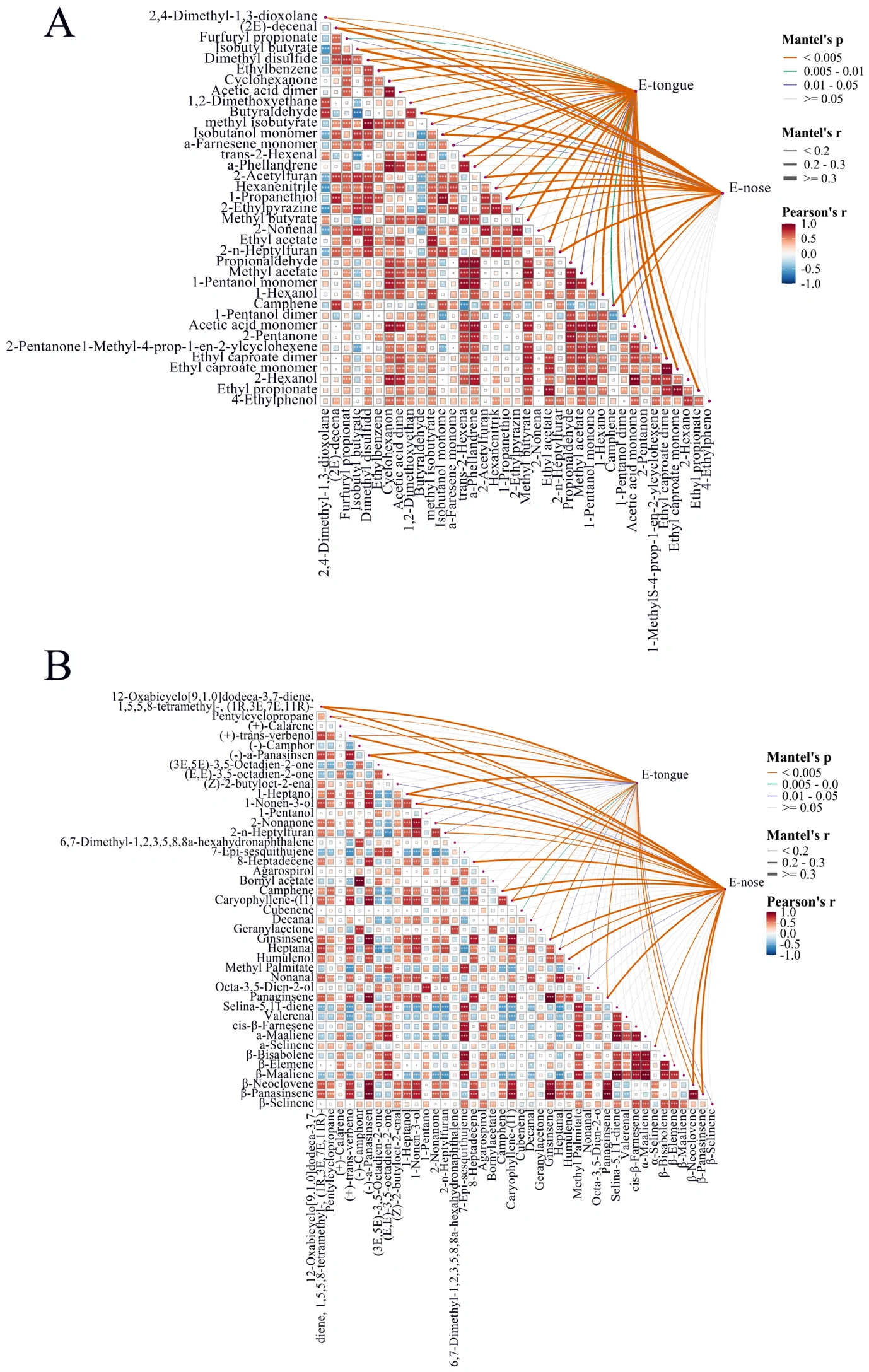
Correlation analysis and comparative overview of VOCs. (**A**,**B**) Heatmaps showing the correlation between key differential VOCs (VIPpred > 1.0 from HS–GC–IMS and HS–SPME–GC–MS, respectively) and E-nose and E-tongue sensor response analysis. Asterisks indicate significance levels: * *p* < 0.05, ** *p* < 0.01, *** *p* < 0.001.

Figure 7A shows that core GC-IMS candidate discriminants, including 1-propanethiol, propionaldehyde, methyl acetate, and acetic acid, were significantly positively correlated with electronic nose sensor responses (*p* < 0.01) and were also significantly correlated with electronic tongue taste signals. Most volatile components in this group were positively correlated with one another, reflecting synergistic variation in small-molecule volatile flavor compounds. In Figure 7B, HS–SPME–GC–MS characteristic components, such as β-bisabolene, β-elemene, β-maaliene, and other terpenoids, were also significantly associated with electronic nose and electronic tongue signals, mutually confirming the HS–GC–IMS conclusions. This consistency indicates that the differential volatile components detected by HS–GC–IMS and HS–SPME–GC–MS together constitute the chemical basis for electronic sensory identification of American ginseng flavor differences.

In contrast, the candidate discriminants obtained by the two methods were significantly correlated with electronic tongue signals, but the overall correlation strength was weaker than that with the electronic nose, indicating that volatile aroma substances may form a coupling relationship with the taste characteristics of American ginseng through the co-regulation of postnasal olfaction and metabolism. The correlation patterns of key flavor components and electronic sensory signals in Figure 7A,B were highly consistent. A small number of negative correlations were observed among components in the two characteristic volatile compound sets, suggesting that some flavor compounds exhibited substrate competition in their biosynthetic pathways. From a sensory perspective, the differential volatile components identified here may contribute to the quality differences in American ginseng from the three regions. Terpenoids such as camphene, α-phellandrene, β-elemene, and α-farnesene generally impart woody, spicy, and herbal odor notes, and their higher abundance in CAN and SD samples may be associated with a stronger characteristic ginseng aroma. Aldehydes and alcohols, such as hexanal, nonanal, 1-hexanol, and 1-pentanol, contribute green and fatty odor notes, whereas pyrazines and furans contribute roasted and nutty notes; the enrichment of these compounds in JL samples may confer a milder, more roasted aroma. In terms of taste, the significant correlation between these volatiles and the E-tongue signals suggests that aroma compounds may influence taste perception through postnasal olfaction, thereby contributing to the observed differences in bitterness, astringency, richness, and sourness. However, the present study focused on the correlation between candidate discriminants and electronic sensory signals, and the direct relationship between these compounds and the overall quality of American ginseng requires further verification by sensory evaluation and bioactivity assays. In summary, HS–GC–IMS and HS–SPME–GC–MS, combined with electronic nose and electronic tongue technologies, can complementarily characterize the overall flavor quality of American ginseng at different molecular scales.

## 4. Conclusions

This study used American ginseng from three regions as experimental materials and combined E-nose and E-tongue bionic sensory techniques, HS–GC–IMS, HS–SPME–GC–MS, and multivariate statistical analysis to explore flavor differences and the chemical basis of American ginseng from different origins. The electronic nose and electronic tongue combined with the PLS–DA model effectively distinguished the three producing areas of American ginseng, and key discriminating sensors and taste indicators were screened, confirming the feasibility of using electronic senses for rapid tracing of American ginseng producing areas. HS–GC–IMS and HS–SPME–GC–MS provided complementary detection, focusing on small-molecule polar volatiles and sesquiterpene components.

Compared with previous studies on American ginseng geographical traceability [3,13,17], many prior investigations relied on a single volatile detection technique or focused on nonvolatile ginsenoside markers. Ref. [17] used an electronic nose coupled with GC–MS to distinguish American ginseng samples but did not employ GC–IMS for highly volatile components. Ref. [13] targeted ginsenosides, which are not flavor-active volatile substances. Although Ref. [3] performed metabolomic characterization of American ginseng, it did not combine two complementary volatile profiling platforms to cover analytes with a wide volatility range. In this study, we integrated electronic nose, HS–GC–IMS and HS–SPME–GC–MS to obtain a comprehensive volatile fingerprint, capturing both highly volatile small molecules and medium-to-low volatility terpenoids. It is worth emphasizing that similar variation trends at the chemical class level cannot be regarded as cross-validation for individual candidate markers. Only a limited number of terpenoids and carbonyl compounds were identified by both chromatographic platforms and showed consistent batch-level abundance changes across the three groups. Most discriminant volatile candidates were detected exclusively by one platform. Both methods demonstrated that terpenoids were the main volatile components of American ginseng, and 37 and 42 candidate discriminants were screened by OPLS–DA (VIPpred > 1), respectively. Pearson correlation and Mantel test results showed that these volatile components were significantly correlated with electronic nose signals, constituting the chemical basis for electronic nose identification of origin-related aroma differences. The significant but weak associations with electronic tongue taste signals indicated that aroma substances may exert a coupling effect on taste through the co-regulation of postnasal olfaction and metabolism. Most candidate discriminants exhibited co-accumulation, whereas a few components showed pathway substrate competition.

First, the VIP > 1 threshold used in this study serves as a model-dependent importance measure rather than a formal statistical test. Second, the candidate discriminants (37 and 42 features) were selected from the same discovery dataset used to construct the OPLS–DA models. Due to the limited sample size and the scope of this study, independent external validation, resampling stability assessment, and formal univariate statistical comparisons with multiple comparison corrections were not performed. Therefore, these features should be regarded as candidate discriminants for origin discrimination rather than a fully validated marker panel. Future studies with larger independent cohorts and targeted chemical validation are necessary to confirm their origin specificity and to develop a parsimonious marker panel.

It should be noted that the term ‘origin’ in this study refers to the assigned geographical source of the sampled lots. Because the Canadian material was obtained through commercial channels, its post-harvest history (e.g., drying, storage, and transport) may not be strictly identical to that of the freshly harvested SD and JL samples. Therefore, the observed flavor differences cannot be strictly attributed to geographical mechanisms *per se*, but rather to differences among the sampled lots assigned to these origins.

In summary, an electronic sensory–chemometric flavor evaluation system for American ginseng was constructed, the flavor differentiation characteristics of the sampled origins were clarified, and several volatile characteristic markers were screened. This provides a theoretical reference for flavor fingerprint establishment, origin-based classification, and quality evaluation of commercial American ginseng. Future studies with larger independent cohorts and appropriately matched materials are necessary to confirm the origin specificity of these markers and to further analyze the flavor formation mechanism by incorporating non-volatile active substances.

## Data Availability

The original contributions presented in the study are included in the article/Supplementary Materials; further inquiries can be directed to the corresponding authors.

## Supplementary Materials

The following supporting information can be downloaded at https://www.mdpi.com/article/10.3390/foods15214011/s1, Table S1: Source of American ginseng samples from different origins. Table S2: The performance description of PEN 3 E-nose; Table S3: Response characteristics of SA402B E-tongue sensor electrodes; Table S4: Types of VOCs of American ginseng from different origins detected by HS–GC–IMS; Table S5: Types of VOCs of American ginseng from different origins detected by HS–SPME–GC–MS. Figure S1: Permutation test with 200 permutations. Figure S2: Representative HS–SPME–GC–MS total ion chromatograms (TICs) of American ginseng samples from three geographical origins.

## Abbreviations

The following abbreviations are used in this manuscript:

CAN	Canada
E-nose	Electronic nose
E-tongue	Electronic tongue
GC–IMS	Gas chromatography–ion mobility spectrometry
GC–MS	Gas chromatography–mass spectrometry
JL	Jilin
OPLS–DA	Orthogonal partial least squares discriminant analysis
PCA	Principal component analysis
PLS–DA	Partial least squares discriminant analysis
RMSE	Root mean square error
RSD	Relative standard deviation
SD	Shandong
SPME	Solid-phase microextraction
TIC	Total ion chromatogram
VIP	Variable importance in projection
VOCs	Volatile organic compounds
