# Supplementary material for: Integrated E-Nose, E-Tongue, HS–GC–IMS, and HS–SPME–GC–MS with Multivariate Statistics for Flavor Discrimination and Volatile Marker Screening of American Ginseng from Different Origins
Source: Foods. 2026 Nov 9;15(21):4011. doi: 10.3390/foods15214011 (PMC13762771; doi:10.3390/foods15214011)
Supplement: Supplementary file 1 [file foods-15-04011-s001.zip › Supplementary Materials.pdf]

Table S1 Source of American ginseng samples from different origins.

Geography Label	ID	Origins.	Harvest year	Cultivation age	Root part
SD	SD-1	Rongcheng City, Weihai City, Shandong Province, China	2025	4 years	Main root
SD	SD-2	Rongcheng City, Weihai City, Shandong Province, China	2025	4 years	Main root
SD	SD-3	Rongcheng City, Weihai City, Shandong Province, China	2025	4 years	Main root
SD	SD-4	Wendeng District, Weihai City, Shandong Province, China	2025	4 years	Main root
SD	SD-5	Wendeng District, Weihai City, Shandong Province, China	2025	4 years	Main root
SD	SD-6	Wendeng District, Weihai City, Shandong Province, China	2025	4 years	Main root
SD	SD-7	Wendeng District, Weihai City, Shandong Province, China	2025	4 years	Main root
SD	SD-8	Wendeng District, Weihai City, Shandong Province, China	2025	4 years	Main root
SD	SD-9	Wendeng District, Weihai City, Shandong Province, China	2025	4 years	Main root
SD	SD-10	Wendeng District, Weihai City, Shandong Province, China	2025	4 years	Main root
SD	SD-11	Rongcheng City, Weihai City, Shandong Province, China	2025	4 years	Main root
SD	SD-12	Wendeng District, Weihai City, Shandong Province, China	2025	4 years	Main root
SD	SD-13	Wendeng District, Weihai City, Shandong Province, China	2025	4 years	Main root
SD	SD-14	Wendeng District, Weihai City, Shandong Province, China	2025	4 years	Main root
SD	SD-15	Wendeng District, Weihai City, Shandong Province, China	2025	4 years	Main root
SD	SD-16	Wendeng District, Weihai City, Shandong Province, China	2025	4 years	Main root
SD	SD-17	Wendeng District, Weihai City, Shandong Province, China	2025	4 years	Main root
SD	SD-18	Wendeng District, Weihai City, Shandong Province, China	2025	4 years	Main root
SD	SD-19	Wendeng District, Weihai City, Shandong Province, China	2025	4 years	Main root
SD	SD-20	Wendeng District, Weihai City, Shandong Province, China	2025	4 years	Main root
JL	JL-1	Wanliang Town, Baishan City, Jilin Province, China	2025	4 years	Main root
JL	JL-2	Fusong County, Baishan City, Jilin Province, China	2025	4 years	Main root

JL	JL-3	Fusong County, Baishan City, Jilin Province, China	2025	4 years	Main root
JL	JL-4	Yanbian Korean Autonomous Prefecture, Jilin Province, China	2025	4 years	Main root
JL	JL-5	Tonghua County, Tonghua City, Jilin Province, China	2025	4 years	Main root
JL	JL-6	Fusong County, Baishan City, Jilin Province, China	2025	4 years	Main root
JL	JL-7	Tonghua County, Tonghua City, Jilin Province, China	2025	4 years	Main root
JL	JL-8	Fusong County, Baishan City, Jilin Province, China	2025	4 years	Main root
JL	JL-9	Fusong County, Baishan City, Jilin Province, China	2025	4 years	Main root
JL	JL-10	Fusong County, Baishan City, Jilin Province, China	2025	4 years	Main root
JL	JL-11	Fusong County, Baishan City, Jilin Province, China	2025	4 years	Main root
JL	JL-12	Fusong County, Baishan City, Jilin Province, China	2025	4 years	Main root
JL	JL-13	Fusong County, Baishan City, Jilin Province, China	2025	4 years	Main root
JL	JL-14	Fusong County, Baishan City, Jilin Province, China	2025	4 years	Main root
JL	JL-15	Fusong County, Baishan City, Jilin Province, China	2025	4 years	Main root
JL	JL-16	Fusong County, Baishan City, Jilin Province, China	2025	4 years	Main root
JL	JL-17	Tonghua County, Tonghua City, Jilin Province, China	2025	4 years	Main root
JL	JL-18	Tonghua County, Tonghua City, Jilin Province, China	2025	4 years	Main root
JL	JL-19	Fusong County, Baishan City, Jilin Province, China	2025	4 years	Main root
JL	JL-20	Yanbian Korean Autonomous Prefecture, Jilin Province, China	2025	4 years	Main root
CAN	CAN-1	Ontario, Canada	2025	4 years	Main root
CAN	CAN-2	Ontario, Canada	2025	4 years	Main root
CAN	CAN-3	Ontario, Canada	2025	4 years	Main root
CAN	CAN-4	Ontario, Canada	2025	4 years	Main root
CAN	CAN-5	Ontario, Canada	2025	4 years	Main root
CAN	CAN-6	Ontario, Canada	2025	4 years	Main root
CAN	CAN-7	Ontario, Canada	2025	4 years	Main root
CAN	CAN-8	Ontario, Canada	2025	4 years	Main root

CAN	CAN-9	Ontario, Canada	2025	4 years	Main root
CAN	CAN-10	Ontario, Canada	2025	4 years	Main root
CAN	CAN-11	Ontario, Canada	2025	4 years	Main root
CAN	CAN-12	Ontario, Canada	2025	4 years	Main root
CAN	CAN-13	Ontario, Canada	2025	4 years	Main root
CAN	CAN-14	Ontario, Canada	2025	4 years	Main root
CAN	CAN-15	Ontario, Canada	2025	4 years	Main root
CAN	CAN-16	Ontario, Canada	2025	4 years	Main root
CAN	CAN-17	Ontario, Canada	2025	4 years	Main root
CAN	CAN-18	Ontario, Canada	2025	4 years	Main root
CAN	CAN-19	Ontario, Canada	2025	4 years	Main root
CAN	CAN-20	Ontario, Canada	2025	4 years	Main root

Table S2 The performance description of PEN 3 E-nose.

NO.	Sensor name	Performance description
S1	W1C	Aromatic constituents, benzene
S2	W5S	High sensitivity and sensitive to nitrogen oxides
S3	W3C	Sensitive aroma, ammonia
S4	W6S	Mainly selective for hydrides
S5	W5C	Short-chain alkane aromatic component
S6	W1S	Sensitive to methyl
S7	W1W	Sensitive to sulfides
S8	W2S	Sensitive to alcohols, aldehydes and ketones
S9	W2W	Aromatic ingredients, sensitive to organic sulfides
S10	W3S	Sensitive to long-chain alkanes

Table S3. Response characteristics of SA402B E-tongue sensor electrodes.

**Electrode code**	**Corresponding taste attribute**
CA0	Sourness
C00	Bitterness
AE1	Astringency
UG	Umami
GL1	Sweetness
Aftertaste- B	Bitterness aftertaste
Aftertaste- A	Astringency aftertaste

Table S4 Types of VOCs of American ginseng from different origins detected by HS−GC−IMS.

NO.	Compound	CAS	Formula	MW	RI	Rt [sec]	Dt [a.u.]
1	α-Farnesene dimer	502-61-4	$C_{15}H_{24}$	204.4	1758.7	2927.313	1.48804
2	α-Farnesene monomer	502-61-4	$C_{15}H_{24}$	204.4	1758.9	2928.679	1.4336
3	Isoamyl benzoate	94-46-2	$C_{12}H_{16}O_2$	192.3	1406.3	1217.174	1.47753
4	2-n-Heptylfuran*	3777-71-7	$C_{11}H_{18}O$	166.3	1226.2	791.891	1.40814
5	Butyl isovalerate	109-19-3	$C_9H_{18}O_2$	158.2	1051.7	539.909	1.38144
6	(2E)-decenal	3913-81-3	$C_{10}H_{18}O$	154.3	1614.2	2042.376	1.47228
7	Furfuryl propionate	623-19-8	$C_8H_{10}O_3$	154.2	1597	1957.025	1.47228
8	Isobutyl butyrate	539-90-2	$C_8H_{16}O_2$	144.2	1147.8	663.112	1.33329
9	Ethyl caproate dimer	123-66-0	$C_8H_{16}O_2$	144.2	1244.6	824.673	1.79943
10	Ethyl caproate monomer	123-66-0	$C_8H_{16}O_2$	144.2	1247.1	829.224	1.34029
11	Mesifurane	4077-47-8	$C_7H_{10}O_3$	142.2	1062.4	551.384	1.20446
12	2-Nonanone*	821-55-6	$C_9H_{18}O$	142.2	1400	1198.147	1.4101
13	2-Nonenal*	18829-56-6	$C_9H_{16}O$	140.2	1559.4	1782.16	1.41251
14	2-pentyl furan*	3777-69-3	$C_9H_{14}O$	138.2	1243.1	821.929	1.25258
15	α-Phellandrene	99-83-2	$C_{10}H_{16}$	136.2	1199.2	745.898	1.69083
16	Camphene*	79-92-5	$C_{10}H_{16}$	136.2	1074.1	564.21	1.21851
17	2,3,5,6-Tetramethylpyrazine*	1124-11-4	$C_8H_{12}N_2$	136.2	1485.2	1481.35	1.22049
18	1-Methyl-4-prop-1-en-2-ylcyc lohexene monomer	138-86-3	$C_{10}H_{16}$	136.2	1206.8	758.601	1.21781
19	α-Terpinene	99-86-5	$C_{10}H_{16}$	136.2	1129.6	635.954	1.21695
20	Ethyl 2-methylbutyrate	7452-79-1	$C_7H_{14}O_2$	130.2	1037.5	525.151	1.66581
21	Methyl hexanoate*	106-70-7	$C_7H_{14}O_2$	130.2	1179.4	713.046	1.28312
22	6-Methyl-5-hepten-2-one	110-93-0	$C_8H_{14}O$	126.2	1349.3	1055.896	1.1774

23	trans-2-Octenal	2548-87-0	$C_8H_{14}O$	126.2	1440.2	1324.179	1.33643
24	4-Ethylphenol	123-07-9	$C_8H_{10}O$	122.2	1216.4	774.808	1.19449
25	1-Heptanol*	111-70-6	$C_7H_{16}O$	116.2	1449.8	1356.446	1.40164
26	2-Heptenal dimer	18829-55-5	$C_7H_{12}O$	112.2	1333.4	1015.124	1.66925
27	2-Heptenal monomer	18829-55-5	$C_7H_{12}O$	112.2	1334.5	1017.842	1.25673
28	2-Acetylfuran	1192-62-7	$C_6H_6O_2$	110.1	1514.8	1594.804	1.43424
29	2-Ethylpyrazine	13925-00-3	$C_6H_8N_2$	108.1	1302.2	939.217	1.51735
30	Ethylbenzene	100-41-4	C_8H_{10}	106.2	1134.3	642.818	1.44514
31	Benzaldehyde*	100-52-7	C_7H_6O	106.1	1539.5	1695.768	1.15166
32	1-Hexanol dimer*	111-27-3	$C_6H_{14}O$	102.2	1369.7	1111.166	1.64149
33	2-Hexanol	626-93-7	$C_6H_{14}O$	102.2	1222.5	785.321	1.27659
34	2,4-Dimethyl-1,3-dioxolane	544-55-0	$C_5H_{10}O_2$	102.1	695.8	282.984	1.06654
35	methyl isobutyrate	547-63-7	$C_5H_{10}O_2$	102.1	668.8	269.626	1.13935
36	Methyl butyrate	623-42-7	$C_5H_{10}O_2$	102.1	997.6	485.7	1.44506
37	Ethyl propionate	105-37-3	$C_5H_{10}O_2$	102.1	971.2	463.275	1.44951
38	Isovaleric acid	503-74-2	$C_5H_{10}O_2$	102.1	823.9	355.894	1.50167
39	3-Methyl-2-pentanone	565-61-7	$C_6H_{12}O$	100.2	1013.5	500.99	1.4611
40	2,3-Pentadione dimer	600-14-6	$C_5H_8O_2$	100.1	1034.3	521.864	1.29839
41	Ethyl acrylate	140-88-5	$C_5H_8O_2$	100.1	1007.2	494.874	1.40762
42	Cyclohexanone	108-94-1	$C_6H_{10}O$	98.1	1309.1	955.306	1.45577
43	trans-2-Hexenal monomer	505-57-7	$C_6H_{10}O$	98.1	1232.7	803.28	1.18142
44	2-Hexenal	505-57-7	$C_6H_{10}O$	98.1	1221.5	783.569	1.51636
45	Hexanenitrile	628-73-9	$C_6H_{11}N$	97.2	1307.1	950.595	1.58573
46	Dimethyl disulfide	624-92-0	$C_2H_6S_2$	94.2	753.2	313.595	1.13678
47	1,2-Dimethoxyethane	110-71-4	$C_4H_{10}O_2$	90.1	937.4	436.093	1.31223
48	1-Pentanol dimer*	71-41-0	$C_5H_{12}O$	88.1	1262.9	858.809	1.24871

49	1-Pentanol monomer*	71-41-0	$C_5H_{12}O$	88.1	1262.9	858.809	1.24871
50	2-Methyl propanoic acid dimer	79-31-2	$C_4H_8O_2$	88.1	1592.7	1936.208	1.37084
51	2-Methyl propanoic acid monomer	79-31-2	$C_4H_8O_2$	88.1	1593.8	1941.412	1.15166
52	Butyric acid dimer	107-92-6	$C_4H_8O_2$	88.1	1647.7	2220.364	1.37266
53	Butyric acid monomer	107-92-6	$C_4H_8O_2$	88.1	1647.8	2220.953	1.15499
54	Valeraldehyde	110-62-3	$C_5H_{10}O$	86.1	934.4	433.715	1.42207
55	2-Pentanone	107-87-9	$C_5H_{10}O$	86.1	994.1	482.642	1.36304
56	Crotonic Acid	107-93-7	$C_4H_6O_2$	86.1	824.8	356.451	1.33464
57	3-Methyl-2-butenal	107-86-8	C_5H_8O	84.1	1214.6	771.742	1.36149
58	Cyclopentanone	120-92-3	C_5H_8O	84.1	1204.7	755.097	1.10958
59	1-Propanethiol	107-03-9	C_3H_8S	76.2	842.5	367.927	1.35765
60	Ethyl acetate	79-20-9	$C_3H_6O_2$	74.1	857.1	377.653	1.1939
61	Methyl acetate	79-20-9	$C_3H_6O_2$	74.1	857.1	377.653	1.1939
62	Isobutanol dimer	78-83-1	$C_4H_{10}O$	74.1	1109	606.41	1.38052
63	Isobutanol monomer	78-83-1	$C_4H_{10}O$	74.1	1106.9	603.514	1.18479
64	1-Butanol monomer	71-36-3	$C_4H_{10}O$	74.1	1160.5	682.823	1.19725
65	Butyraldehyde	123-72-8	C_4H_8O	72.1	892.1	402.116	1.28069
66	Acetic acid dimer	64-19-7	$C_2H_4O_2$	60.1	1495.8	1520.902	1.16253
67	Acetic acid monomer	64-19-7	$C_2H_4O_2$	60.1	1497.4	1527.148	1.05384
68	Propionaldehyde	123-38-6	C_3H_6O	58.1	823.4	355.568	1.14582

*: Volatile compounds detected by both HS–GC–IMS and HS–SPME–GC–MS.

Table S5 Types of VOCs of American ginseng from different origins detected by HS−SPME−GC−MS.

No.	RT	RSI	SI	Compound	**CAS**	Formula
1	4.39	887	870	1-Pentano*	71-41-0	$C_5H_{12}O$
2	4.77	950	939	(S,S)-Butane-2,3-diol	19132-06-0	$C_4H_{10}O_2$
3	5.02	882	881	Hexanal	66-25-1	$C_6H_{12}O$
4	6.73	893	891	1-Hexanol*	111-27-3	$C_6H_{14}O$
5	7.26	854	840	4-Methyl-2-Hexanone	105-42-0	$C_7H_{14}O$
6	7.53	923	918	Heptanal	111-71-7	$C_7H_{14}O$
7	8.12	948	942	Methyl Hexanoate*	106-70-7	$C_7H_{14}O_2$
8	8.21	918	902	α-Thujene	2867-05-2	$C_{10}H_{16}$
9	8.83	945	908	Camphene*	79-92-5	$C_{10}H_{16}$
10	8.98	947	911	(Z)-2-Heptenal	57266-86-1	$C_7H_{12}O$
11	9.11	941	919	Benzaldehyde*	100-52-7	C_7H_6O
12	9.35	902	897	1-Heptanol*	111-70-6	$C_7H_{16}O$
13	9.57	896	886	β-Pinene	127-91-3	$C_{10}H_{16}$
14	9.62	914	896	1-Octen-3-ol	3391-86-4	$C_8H_{16}O$
15	9.82	890	845	Hexanoic acid	142-62-1	$C_6H_{12}O_2$
16	9.89	911	890	2-pentylfuran*	3777-69-3	$C_9H_{14}O$
17	10.22	903	898	Octanal	124-13-0	$C_8H_{16}O$
18	10.81	917	893	o-Cymene	527-84-4	$C_{10}H_{14}$
19	10.91	941	926	2-Ethylhexan-1-ol	104-76-7	$C_8H_{18}O$
20	10.94	908	896	Sylvestrene	1461-27-4	$C_{10}H_{16}$
21	11.13	904	904	Octa-3,5-Dien-2-ol	69668-82-2	$C_8H_{14}O$
22	11.28	930	925	Benzeneacetaldehyde	122-78-1	C_8H_8O
23	11.96	902	859	(3E,5E)-3,5-Octadien-2-one	30086-02-3	$C_8H_{12}O$

24	12.03	911	891	Pentylcyclopropane	2511-91-3	C_8H_{16}
25	12.11	883	856	Heptanoic acid	111-14-8	$C_7H_{14}O_2$
26	12.35	904	903	2,3,5,6-Tetramethylpyrazine*	1124-11-4	$C_8H_{12}N_2$
27	12.52	926	908	2-Nonanone*	821-55-6	$C_9H_{18}O$
28	12.57	865	842	(E,E)-3,5-octadien-2-one	38284-27-4	$C_8H_{12}O$
29	12.88	901	891	Nonanal	124-19-6	$C_9H_{18}O$
30	13.35	916	910	Methyl octanoate	111-11-5	$C_9H_{18}O_2$
31	13.96	950	933	trans-verbenol	1820-09-3	$C_{10}H_{16}O$
32	14	951	922	Camphor	464-48-2	$C_{10}H_{16}O$
33	14.27	915	913	2-Nonenal*	18829-56-6	$C_9H_{16}O$
34	14.42	834	816	Borneol	10385-78-1	$C_{10}H_{18}O$
35	14.44	916	908	2-sec-Butyl-3-methoxypyrazine	24168-70-5	$C_9H_{14}N_2O$
36	14.8	899	869	1-Nonen-3-ol	21964-44-3	$C_9H_{18}O$
37	15.06	917	902	2-n-Heptylfuran*	3777-71-7	$C_{11}H_{18}O$
38	15.3	920	918	Dodecane	112-40-3	$C_{12}H_{26}$
39	15.41	899	889	Decanal	112-31-2	$C_{10}H_{20}O$
40	15.71	835	823	6,7-Dimethyl-1,2,3,5,8,8a-hexahydronaphthalene	107914-92-1	$C_{12}H_{18}$
41	17.7	912	909	Bornyl acetate	76-49-3	$C_{12}H_{20}O_2$
42	18.59	915	892	Panaxene	871660-95-6	$C_{15}H_{24}$
43	19.51	926	892	Panaginsene	871660-96-7	$C_{15}H_{24}$
44	20.15	920	898	α-Cubebene	17699-14-8	$C_{15}H_{24}$
45	20.23	932	929	Ginsinsene	871660-97-8	$C_{15}H_{24}$
46	20.51	917	904	γ-nonalactone	104-61-0	$C_9H_{16}O_2$
47	21.17	901	890	(Z)-2-butyloct-2-enal	13019-16-4	$C_{12}H_{22}O$
48	21.25	832	813	β-Maaliene	489-29-2	$C_{15}H_{24}$
49	21.49	909	904	Copaene	3856-25-5	$C_{15}H_{24}$

50	21.99	914	913	β-Panasinsene	56684-97-0	$C_{15}H_{24}$
51	22	856	840	α-isocomene	65372-78-3	$C_{15}H_{24}$
52	22.23	913	912	β-Elemene	515-13-9	$C_{15}H_{24}$
53	22.38	915	821	8-Heptadecene	2579-04-6	$C_{17}H_{34}$
54	24.15	846	843	Caryophyllene-(I1)	22567-17-5	$C_{15}H_{24}$
55	24.67	924	921	Calarene	17334-55-3	$C_{15}H_{24}$
56	24.88	907	899	α-Maaliene	489-28-1	$C_{15}H_{24}$
57	25.01	873	831	(1R,8aR)-1,8a-dimethyl-7-propan-2-ylidene-1,2,6,8-tetrahydronaphthalene	27840-40-0	$C_{15}H_{22}$
58	25.67	939	923	Selina-5,11-diene	52026-55-8	$C_{15}H_{24}$
59	25.83	847	822	Geranylacetone	3796-70-1	$C_{13}H_{22}O$
60	26.11	934	931	α-Panasinsen	56633-28-4	$C_{15}H_{24}$
61	26.28	892	890	cis-β-Farnesene	28973-97-9	$C_{15}H_{24}$
62	27.84	910	906	β-Neoclovene	56684-96-9	$C_{15}H_{24}$
63	28.52	899	885	Curcumene	644-30-4	$C_{15}H_{22}$
64	28.71	901	892	Sesquisabinene	58319-04-3	$C_{15}H_{24}$
65	28.97	889	874	β-Selinene	17066-67-0	$C_{15}H_{24}$
66	29.68	908	890	α-Selinene	473-13-2	$C_{15}H_{24}$
67	29.79	916	883	7-Epi-sesquithujene	159407-35-9	$C_{15}H_{24}$
68	30	911	896	α-Muurolene	31983-22-9	$C_{15}H_{24}$
69	30.39	896	894	γ-Himachalene	53111-25-4	$C_{15}H_{24}$
70	30.92	917	910	β-Bisabolene	495-61-4	$C_{15}H_{24}$
71	31.27	920	899	Cubebol	23445-02-5	$C_{15}H_{26}O$
72	31.56	913	902	Naphthalene, 1,2,3,5,6,8a-hexahydro-4,7-dimethyl-1-(1-methylethyl)-, (1S-cis)-	16729-01-4	$C_{15}H_{24}$
73	32.31	945	848	Cubenene	29837-12-5	$C_{15}H_{24}$
74	32.86	827	814	α-Agarofuran	5956-12-7	$C_{15}H_{24}O$

75	33.67	891	867	Nerolidol	40716-66-3	$C_{15}H_{26}O$
76	34.03	840	807	Epiglobulol	88728-58-9	$C_{15}H_{26}O$
77	34.11	911	908	Spathulenol	77171-55-2	$C_{15}H_{24}O$
78	34.5	943	931	Globulol	489-41-8	$C_{15}H_{26}O$
79	34.81	839	819	Isoaromadendrene epoxide	499134-59-7	$C_{15}H_{24}O$
80	35.26	823	801	α-Patchoulene	560-32-7	$C_{15}H_{24}$
81	35.53	913	903	HumuleneepoxideII	19888-34-7	$C_{15}H_{24}O$
82	35.84	909	899	Neointermedeol	5945-72-2	$C_{15}H_{26}O$
83	36.15	826	811	Oxacyclotetradeca-4,11-diyne	6568-32-7	$C_{13}H_{18}O$
84	36.28	899	889	Humulenol	19888-00-7	$C_{15}H_{24}O$
85	36.61	952	901	Agarospirol	1460-73-7	$C_{15}H_{26}O$
86	37.46	841	828	Zingiberenol	58334-55-7	$C_{15}H_{26}O$
87	38.42	820	817	Valerenal	4176-16-3	$C_{15}H_{22}O$
88	39.92	913	913	Methyl Palmitate	112-39-0	$C_{17}H_{34}O_2$
89	40.37	887	884	Ethyl palmitate	628-97-7	$C_{18}H_{36}O_2$

*: Volatile compounds detected by both HS–GC–IMS and HS–SPME–GC–MS.

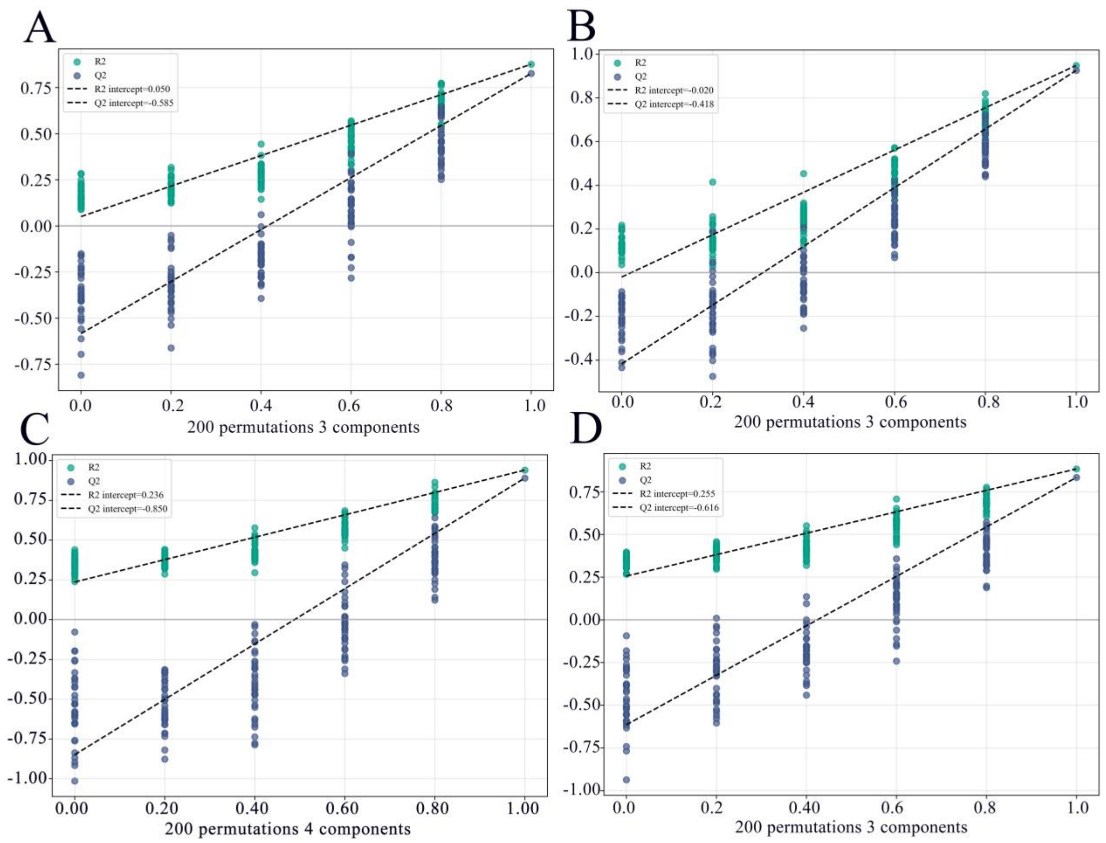

Figure S1 Permutation test with 200 permutations. (A) Permutation test of PLS−DA for E-nose. (B) Permutation test of PLS−DA for E-tounge. (C) Permutation test of OPLS−DA for HS−GC−IMS. (D) Permutation test of OPLS−DA for HS−SPME−GC−MS.

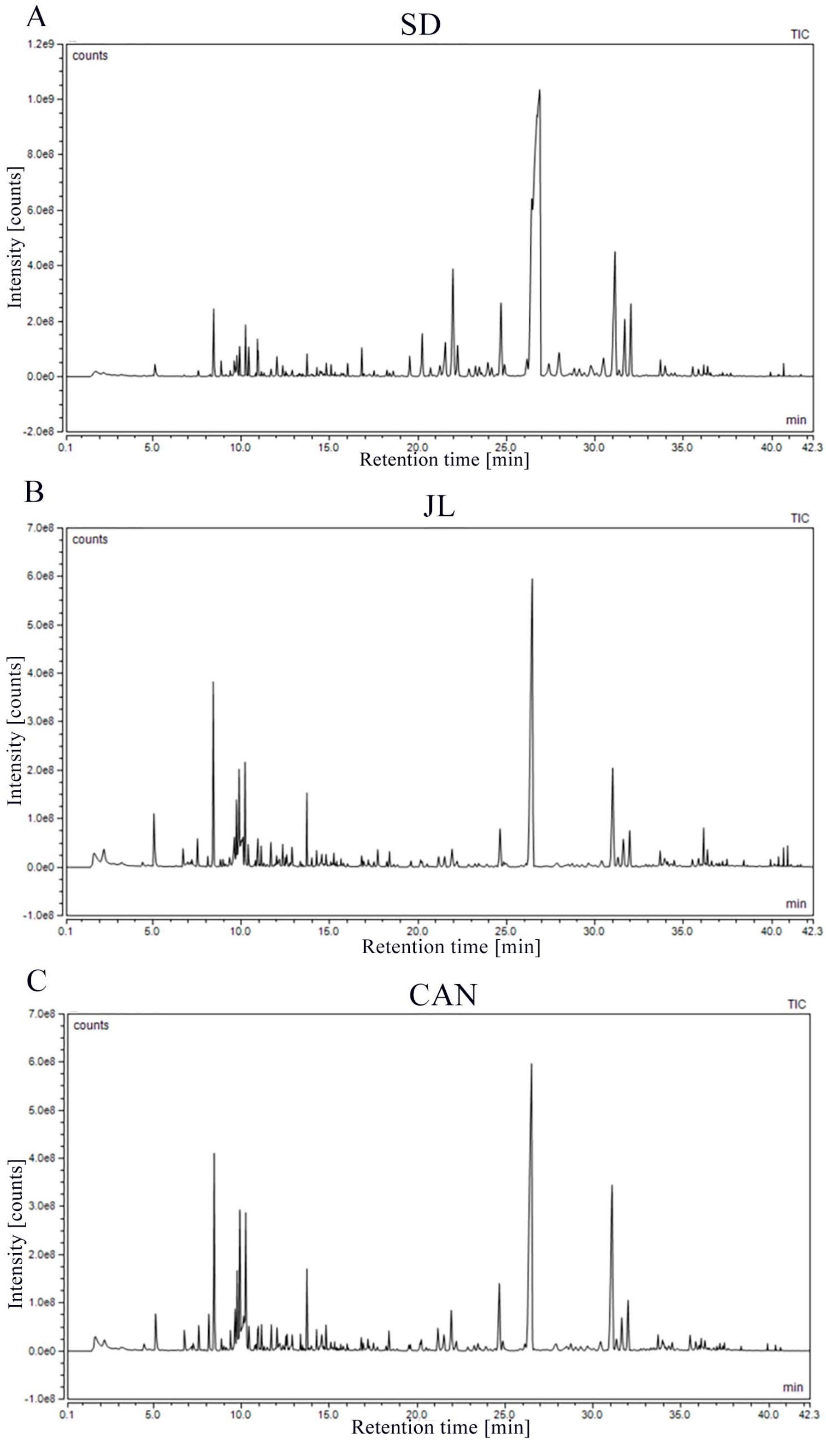

Figure S2 Representative HS−SPME−GC−MS total ion chromatograms (TICs) of American ginseng samples from three geographical origins. (A) Shandong (SD, China); (B) Jilin (JL, China); (C) Canada (CAN). The x-axis represents the retention time (min), and the y-axis represents the signal intensity (counts). The total ion current (TIC) mode was used for data acquisition.
